# The Hospitals Association

**Published:** 1888-02-18

**Authors:** Timothy Holmes


### THE HOSPITALS ASSOCIATION: THE RELATION OF THE MEDICAL SCHOOL TO THE HOSPITAL.

By TIMOTHY HOLMES, F.R.C.S.

THERE was a fair attendance on Wednesday evening, the 8th inst., at a meeting of The Hospitals Association, held at St. George's Hospital, Hyde Park, when Mr. Timothy Holmes, F.R.C.S., read a paper on "The Relation of the Medical School to the Hospital." Mr. J. S. BRISTOWE, M.D., F.R.S., the President of the Association, took the chair, and there were present: Lieut.-General Keatinge, Colonel Heygarth (Treasurer of St. George's Hospital), Dr. Ewart, Mr. Wegg Prosser, Mr. Wood Hill, Dr. Mannsell, Mr. Carr-Gomm, Mr. Keith Young, Mr. Ryan, Mr. Cutler, Mr. Bennett, Mr. Myers, Dr. Penrose, Mr. Todd (Secretary of St. George's Hospital), the Rev. J. Marshall, Mr. Mackenzie, Mr. Hayes, Mr. Gattie, Mr. Shields, Mrs. Holme, Mrs. Perry, Miss Robertson, Mrs. Bedingfield, Mrs. Robinson, Miss Mackenzie, and others.

In introducing the lecturer, the CHAIRMAN remarked that he was so well known, particularly within the walls of that institution, that any words from him were needless in presenting to the audience a gentleman who had been for so many years such a distinguished ornament, and so admirable and valued a member of the governing body of St. George's.

Mr. Holmes, who was received with cheers, proceeded to read his paper.

MR. PRESIDENT,—

I must request your indulgence, and that of my audience, on the ground that I have been asked at the last moment to undertake a task which would have been far better performed by Dr. Gilbart-Smith, who was to have treated this subject to-night, had not ill-health and other domestic circumstances rendered it unfortunately impossible for him to fulfil his engagement. In every other respect you have reason to regret the change; but in one particular I may perhaps be able to speak with more freedom than he could, since I am no longer personally interested in the medical school, with which it has been my pride to be connected as student and teacher for now nearly forty years. This long experience has, I hope, qualified me to speak with some knowledge of the subject, though perhaps I cannot claim to be entirely impartial.

I must not forget that I am speaking to a mixed audience, some of whom possibly share in the ignorance, or even prejudice, that so largely prevails among the public on the subject of medical schools—a prejudice which I believe is now rapidly passing away, but of which we see every now and then some half-ludicrous, half-mischievous proofs. It is on account of this mixed character of my audience that I shall say a good deal which to medical hearers would sound trite and matter of course, but which I trust will be pardoned under the circumstances.

A great hospital is a very complex institution, and may for our present purpose be regarded as made up of many departments—a body of governors, to superintend and regulate the whole concern; a financial agency, to raise and expend the necessary money; a medical staff for the actual treatment of the sick; a place for the study of diseases and the improvement of the medical art; a centre for the medical charities of the district, by which their organisation can be controlled and their deficiencies supplemented.

My object in this paper will be to show how great an assistance a medical school is to all these departments of hospital administration, and how greatly the governing body of a hospital can promote the end for which they exist by favouring the growth and extending the reputation of the medical school. Let us first look at the hospital as a society of governors bound together for one great object—the care of the sick poor. I should hardly have thought it necessary to argue that from this society those should not be excluded who know most thoroughly every detail of the system, and whose interests and feelings are most closely bound up with its success, were it not that I believe in some of the older hospitals the plan is still followed by which the governors of the hospital and the officers and members of the medical school are kept distinct bodies. I hope that in characterising such a form of government as antiquated and unreasonable I am not transgressing the bounds of courtesy; but what else can we say of a system which makes a man's knowledge of the institution a formal disqualification for a voice in its management? If it be replied that the medical officers of a hospital have interests both in the hospital and the school which are different from, and may sometimes conflict with, those of the lay element, I fully admit it, and recognize in the fact a reason for having a preponderating lay element in the governing body. But it seems to me a very great mistake, in the interest of the hospital or (which is the same thing) of the patients, to treat the medical staff as if they were merely the servants for that purpose of the lay managers—and if this is unadvisable even in hospitals where there is no medical school, it is doubly to be deprecated where another institution so complicated and peculiar as a school is, and at the same time so useful to the hospital, has also to be considered by the governors. No one who knows anything of our great hospitals but must admit that the school exercises the greatest influence on the hospital; and it seems a truism to say that the affairs will be better managed if those who alone understand them thoroughly have a voice in the deliberations. The other course, in which the lay-governors alone manage, while the medical men can only offer advice when asked, leads to all kinds of favouritism and undue influence, in which the property of the hospital is quite as often, I believe, unduly charged for the benefit of the medical school as the reverse. Open dealing is best in these, as in all other matters, and the affairs of hospitals are, as it seems to me, best managed by an open board or a large committee, on which the medical staff have by right a seat, but in which they are, of course, in a minority, and in which the interests of the medical school are publicly considered, and are promoted whenever they do not conflict with those of the patients. And that is a contingency which will rarely indeed occur.

I have the happiness to-night of speaking in a hospital where this form of government has always prevailed, and I would confidently appeal to both parties—both the medical and lay element—whether the result is not satisfactory to both—whether it does not tend both directly and indirectly to the benefit of the hospital, which is another phrase for the benefit of the patients. Under this plan, a school which from its position could never aspire to rival those of the great central hospitals in numbers, has maintained its grea efficiency and reputation since the days of John Hunter, and has rendered services to the hospital which are always generously admitted by the Board of Governors, and are requited by a constant support and consideration, which it is a grateful duty on every fitting occasion to acknowledge. A hospital, again, is a financial agency for raising and expending the funds required for its maintenance—no light task, at the present time especially. Some of our hospitals were regarded formerly as above the need of subscriptions, and were spoken of as "endowed hospitals"; but with one fortunate exception even these have been visited by the same depression which has fallen so severely on other owners of land, and have been forced to ask the assistance of the charitable public. Now, in seeking that assistance there is no more potent auxiliary than a good medical school. It helps in every way the financial efforts of the lay-governors. Its members and their immediate connections form a respectable proportion of the subscribers; its students ultimately form the greater part of the practitioners of good standing in the neighbourhood; and their recommendations to their patients procure a still larger portion of the subscribers. The public reputation of the school is another great factor in procuring subscriptions, and many of the valuable legacies which form so large a part of hospital property are due to the medical school.

Perhaps the most splendid acquisition which any hospital ever received by legacy is the Atkinson-Morley Hospital at Wimbledon, attached to St. George's. This is called a convalescent hospital, but that name only inadequately describes it. It is really a branch of St. George's in the country, and, besides many other most useful functions, it relieves the town hospital of cases no longer urgent, but not yet fit for dismissal. For this grand addition to our institution we are indebted to the fact that Mr. Morley was originally a student at this hospital. And I do not doubt that the influence of the medical school might be traced in many of the other legacies by which the inadequacy of our subscription list has hitherto happily been supplied.

Not less important is the aid given by the medical school in regulating the expenditure of the funds when raised. There is no more efficient guarantee for real economy in a hospital than the constant presence of intelligent students in the wards. By economy I do not mean small expenditure, but the obtaining efficiency at a reasonable cost. All the trifling deficiencies which often make the whole difference between efficiency and inefficiency are revealed by the constant supervision which a medical school entails, and the constant necessity of supplying what is lacking for the needs of clinical teaching. I am always glad to see an increased expenditure on the items of our accounts headed "Medical and Surgical Expenses," since this means increased activity in the great battle which we are always waging with disease, arms of increased precision, and in all probability an increase in the severity of the cases with which we grapple. Every surgeon knows how much more complicated and how much more expensive the system of dressing wounds has become of late years, but every surgeon as old as I am knows also that this increased expense has been attended by an increase in the safety of our operations, so great that my late friend, Mr. Callender, estimated that amputations did not involve more than a tenth of the mortality which prevailed when he was a student, and the estimate is probably not very wide of the mark. Any wise expenditure which leads to improvement like this is true economy, and such improvement is obviously greatly assisted and promoted by the presence of an efficient medical school.

A hospital may also be regarded as a body of physicians and surgeons united together for the purpose of treating the patients confided to them by the governors. And considered in this light the importance of a medical school to the hospital can hardly be exaggerated. It creates and maintains that *esprit de corps*, that healthy rivalry in good works, which forms so striking a feature of the medical system of London as distinguished from that of Paris. Our great medical schools here are extremely like the various colleges at our Universities. We have our traditions here at St. George's—our great men, whose portraits and busts adorn this room—we are proud of the discoveries of our predecessors and colleagues in the school, and anxious to distinguish ourselves in medicine or surgery, partly to add lustre to it, not merely to aggrandise ourselves. The same, of course, is true of the other schools, and hence arises a contest for priority which is not confined to the schools of this metropolis alone, but which extends to those of other parts of the kingdom also—so that as Guy's is eager to outstrip St. Bartholomew's, so Edinburgh is eager to rival London; and it is doubtless in great measure to this wholesome competition that the extraordinary activity and progress of the British school of medicine and surgery is due—a competition which is wanting in countries where the State supplies the hospitals with medical officers, all bred in the same central school. The *esprit de corps* from which this competition springs is as directly beneficial to the individual hospitals as to the study of medicine in general. The intimate acquaintance which the members of the staff have with each other—the kindly feelings and mutual confidence which naturally spring up amongst men who stand to each other in the pleasant relations of teachers, fellow-students, and pupils, are of immense direct advantage to the patients. Further, the need for clinical instruction is another most important benefit to the sick. Hasty people are apt to think the reverse—to assume that the patient's comfort is interfered with, and his recovery jeopardised by a prying crowd of students. Nothing, I think, is further from the truth. If it were my fortune to be an hospital patient I should pray that I might be treated in a clinical hospital, where my physician would have to make himself minutely acquainted with my case, that he might be able to explain it, and to vindicate his diagnosis and treatment to an intelligent audience of students; or my surgeon would have (as he would here at St. George's) to discuss the case with his colleagues in presence of the students, to bear the free criticism of his fellows, and prove the justice of his proposals before he risked my life in the operating theatre.

As to the interference of students with the patients, I never heard in the long course of my experiences here any complaint of the sort, either made by the patient himself, or discovered by the lay visitors who so zealously and efficiently superintend the working of the hospital, and to whose care both the medical staff and the patients owe so much. But I would not have you think that our London schools are close corporations into which no one is admitted who has not paid his footing and been enrolled in the guild. This, no doubt, was so, at least at some schools, formerly; a student by paying a large fee to one of the surgeons could ensure to himself the offer of a vacancy in time. But all this, like many other old customs which have grown into abuses, is changed now. Neither this nor any other school has any hesitation in going outside its own ranks whenever it may seem necessary for the general well-being; and though such occasions are rare, at least in vigorous and well-managed schools, and so do not interfere with the general *esprit de corps* on which I have laid such stress, instances of them can be produced, I think, from every school in London, and our own has just furnished a conspicuous but by no means the first example. That a hospital is a place for the medical care and treatment of the sick is so obvious that the world is apt to forget that it has another, and not less important object—that of the study of the medical art. I say advisedly not a less important object; nay, I think the progress of medicine is the more important of the two to the human race, for the proportion of the human race who receive relief in their own persons as hospital patients is but small when compared with those who owe their lives, their comfort, their peace of mind, their sanity, to the improvement which is so rapidly going on in the medical art.

Men who, like myself, can look back to about forty years passed in the service of a large hospital can testify to a revolution in the practice of medicine not less remarkable than that which has taken place in mechanical or electrical science. Of course the most wonderful, and to the laity the most intelligible proof of this revolution is the abolition of pain in surgical operations, and this wonderful discovery was no doubt made by private persons of no remarkable medical knowledge, and unconnected with the hospitals or schools. But the diminished mortality of all surgical operations, the astonishing increase of the field of operative surgery, the increase of knowledge of the nature and treatment of fevers, the immense stride which has been made since the commencement of this century in the treatment of mental affections, and above and beyond all (because containing in itself the promise of other advances at which we cannot even guess at present) the foundation and rapid advance of the science of pathology, are only some among the many factors of the advance of medicine which are only possible by combined and disciplined research; and for which the opportunities supplied by large hospitals, and the co-operation of a trained staff of observers in the hospital school, are absolutely necessary.

The somewhat silly controversies which have lately gone on in the press have, perhaps, blinded people in some degree to the real road by which medical progress is effected and men's lives preserved. Much, no doubt, is done by prevention—perhaps more than by attempts at cure; and of these preventive measures the greater part are beyond the sphere of the medical art in its strict sense, though it is, of course, to the medical profession that the public is and must be indebted for its protection from the dangers of bad ventilation and bad drainage, and for the demonstration of the ills which follow in the train of poverty, vice, dissipation, unhealthy employments, and the like. But in matters of disease, strictly speaking, it is well to remember and to get the public to see that all the sound progress which medicine has made in the past, and all that we can hope it will make in the future, rests upon pathology; that therapeutical theories, however ingenious, and study of symptoms, however extensive, have little influence in medicine compared to that knowledge of the causes and effects of disease which is given by pathology. Now the investigations of pathology are becoming constantly more laborious and intricate, and it is only the influence of the great schools of medicine which can prevent such studies from breaking up into a host of specialities, and the medical art from degenerating into a struggle between rival nostrum-mongers—it is only the co-operation, not of the members of one school only, but of all the schools working together, which can elicit the truth from the struggle of rival theories, can test all alleged discoveries by the results of experience, and so push forward the possibly slow but certain advance which we are making towards a more definite and more comprehensive pathology. It is, then, most necessary for a great nation like ours that there should be various independent schools of medicine, supported as such schools can alone be by the resources of hospitals of adequate size.

